# Decentralized Machine Autonomy for Manufacturing Servitization

**DOI:** 10.3390/s22010338

**Published:** 2022-01-03

**Authors:** Matevž Pustišek, Min Chen, Andrej Kos, Anton Kos

**Affiliations:** 1 Faculty of Electrical Engineering, University of Ljubljana, 1000 Ljubljana, Slovenia; andrej.kos@fe.uni-lj.si (A.K.); anton.kos@fe.uni-lj.si (A.K.); 2 School of Computer Science and Technology, Huazhong University of Science and Technology, Wuhan 430074, China; minchen2012@hust.edu.cn

**Keywords:** machine autonomy, servitization, use case, decentralized application, smart contract

## Abstract

Blockchain ecosystems are rapidly maturing and meeting the needs of business environments (e.g., industry, manufacturing, and robotics). The decentralized approaches in industries enable novel business concepts, such as machine autonomy and servitization of manufacturing environments. Introducing the distributed ledger technology principles into the machine sharing and servitization economy faces several challenges, and the integration opens new interesting research questions. Our research focuses on data and event models and secure upgradeable smart contract platforms for machine servitization. Our research indicates that with the proposed approaches, we can efficiently separate on- and off-chain data and assure scalability of the DApp without compromising the trust. We demonstrate that the secure upgradeable smart contract platform, which was adapted for machine servitization, supports the business workflow and, at the same time, assures common identification and authorization of all the participants in the system, including people, devices, and legal entities. We present a hybrid decentralized application (DApp) for the servitization of 3D printing. The solution can be used for or easily adapted to other manufacturing domains. It comprises a modular, upgradeable smart contract platform and off-chain machine, customer and web management, and monitoring interfaces. We pay special attention to the data and event models during the design, which are fundamental for the hybrid data storage and DApp architecture and the responsiveness of off-chain interfaces. The smart contract platform uses a proxy contract to control the access of smart contracts and role-based access control in function calls for blockchain users. We deploy and evaluate the DApp in a consortium blockchain network for performance and privacy. All the actors in the solution, including the machines, are identified by their blockchain accounts and are compeers. Our solution thus facilitates integration with the traditional information-communication systems in terms of the hybrid architectures and security standards for smart contract design comparable to those in traditional software engineering.

## 1. Introduction

Information and communication technologies (ICT) have a paramount role in the digitalization of manufacturing and logistics. Digitalization builds on automation, robotics, and the Internet of Things (IoT) for industrial environments, the extensive use of cloud services, advanced data analytics, and close integration with the existing enterprise resource planning (ERP) systems. Digitalization is mostly based on proven centralized production-grade solutions for maintaining high-quality standards. They enable highly connected machines and smart cloud-based management for remote monitoring and control or preventive maintenance. However, the expectations reach far beyond this. From the business perspective, manufacturing and supply chains are relatively static, driven by long-term agreements, and rely on a high level of trust between participants [1]. Machine-sharing economy principles and servitization impose a mix of new business and technical requirements for future digitalization. The relationships between participants need to be more dynamic, including short-term and ad hoc relationships, and the manufacturing and supply chains need to be coupled even closer. Nevertheless, the mutual trust between the participants cannot always be taken for granted.

A machine-sharing economy enables optimal utilization of machine capacities. These can be offered in a digitized marketplace, and the manufacturers pay for the machines based on their actual use (pay per use). Servitization promises even more complex business arrangements, such as the manufacturing of personalized products, social manufacturing, virtualized factories, and economically autonomous machines. These concepts are motivated by successful examples of sharing economies in other domains (e.g., tourism and transportation). They are also encouraged by clear benefits for the providers and service consumers and the lessons learned in the ICT domain (e.g., from mobile virtual network operators (MVNO) or different virtualized and cloud service providers).

Distributed ledger technology (DLT) is an important enabler for the machine-sharing economy and servitization. It facilitates a trusted transaction recording and a business logic execution environment. It equally supports business, human, and machine participants and assures efficient identification, authentication, and autonomy. Intermediaries or trusted third-party providers are no longer required, as the trust is assured by decentralization and incorporated security mechanisms in DLT. Decentralized applications (DApp), which are based on DLT, can integrate the DLT features with the cloud or other non-DLT services and thus assure interoperability with the existing industrial systems.

However, introducing the DLT principles into the machine-sharing and servitization economy faces several challenges, and the integration opens new and interesting research questions. These include, for example, DLT network performance, interoperability, standardization, scalability, hybrid DApp architectures, and DApp security. In our research, we focus on two of these aspects. We propose a data and event model for an adaptable and scalable DApp and present a secure, upgradable smart contract platform adapted for machine servitization. Both contributions are essential steps for the DApp architectures to progress from the current demonstration phases to the future use in real industrial environments. We designed, developed, and evaluated a decentralized solution for sharing 3D printing resources in our research. In this way, we created an emulation environment to verify the feasibility of the proposed data and event models and the secure smart contract platform, as 3D printers become autonomous entities and are identified by their unique blockchain accounts. They can offer, negotiate, or order printing services from other printers, and 3D printing is just an example use case that can be extended to other manufacturing cases and servitization. The smart contracts are designed to be modular, upgradeable, and updatable. These features assure secure maintenance of the smart contract platform and seamless service operation. We establish a consortium proof-of-authority blockchain network to meet our solution’s security and performance needs. A blockchain-aware, web-based user interface facilitates management and monitoring of the system.

The key contributions of our research presented in this paper are the following:

1. We demonstrate a blockchain-based system where manufacturing machines act as completely autonomous entities which can share or order manufacturing resources from others. Machines do have owners, but they do not (have to) intervene in any machine service-provisioning communications;
2. We define the data and event models for a hybrid on-chain and off-chain decentralized application. They enable efficient splitting of application logic and data storage between the on-chain and off-chain application parts for an effective and scalable solution and integration with legacy systems;
3. A secure modular smart contract platform was designed and developed to support the desired business logic and demonstrated in an autonomous 3D printing service. In designing the data and event models and the smart contract platform, we anticipated the requirements of business-grade decentralized applications, their productization, and seamless extension to other use cases for machine sharing and servitization in manufacturing and supply chains.

The remainder of this paper is organized as follows. Section 2 highlights some recent endeavors on machine autonomy and servitization with blockchains in manufacturing. Section 3 gives the directions toward decentralized applications dealing with the IoT. In Section 4, we declare the use case and involved actors. Section 5 presents the design and development, and Section 6 evaluates a decentralized servitization solution sharing 3D resources among economically autonomous machines. In the Conclusion, we summarize the key findings and outline future research directions.

## 2. Background

Decentralized applications combine the distributed ledger technology (DLT) aspects with web, mobile, or other technologies. This combination is needed to integrate trustworthy decentralized backend application parts, running in DLT networks and with a non-DLT cloud-based backend, either for the mobile and web-based user or machine interfaces. Blockchains (BC) are the most common form of DLT.

DLT-based IoT applications share many aspects with decentralized applications in other application domains. However, some additional requirements and limitations apply due to the nature of the IoT systems. These are, for example, DL network performance, smart contracts, and end-device security, or the participation of constrained IoT devices in a DApp. The design of DApps for the IoT requires careful consideration of the application architecture, specifically defining DL’s meaningful role in the DApp. Initially, DLT and blockchains’ distributed nature was seen as a potential cure for many of the challenges appearing in cloud-centered IoT solutions, including scalability, availability, limited network performance, and cost [2]. However, many of these expectations have been proven to be false, and these challenges are being addressed efficiently without DLT (e.g., virtualization in cloud and networks or edge or fog architectures improving the cloud). We would rather see the key potentials of blockchains in the IoT to provide new features derived from the trusted, decentralized nature of the DLT. These are, for example, the improved trust with no central authorities, full autonomy in device operation and business, seamless M2M transactions and trusted operation through the smart contracts, trustworthy DApps, known data provenance, and fairness through financial incentives [3].

### Blockchain Technologies in Industry and Manufacturing

In [4], the authors conducted a study with 50 experts to examine blockchain technology’s overall impact and role in the machine economy. This study defined and evaluated several projections about the potential role of blockchain technologies in machine autonomy. These projections clearly pointed out the anticipation of expert communities for machine economic autonomy, including independent machine identification, decision making, and value exchange. In terms of technology, key issues are found in blockchain scalability and integration with other enabling technologies, such as the industrial IoT, cloud, and advanced data analytic and AI techniques. Apart from that, their findings point out the relevance of standardization, legislation, and regulatory frameworks for machine economy. These endeavors enable further moves from product-based to service-driven manufacturing scenarios, where BC is one of the key technology drivers for servitization [5]. It enables not only tamper-proof data recording but also a fast and secure decision-making process in critical areas such as ordering, billing, and warranty management and the automation of value networks [6]. Various incumbent organizations and start-ups examined blockchains as the basis of the machine economy. So far, none of the solutions are in a productive status [4]. In [7], the authors evaluated the role of blockchain technology in addressing the critical bottlenecks in business process management. They outlined a blockchain-based framework to select and compose services in open business environments.

One of the first non-financial domains where research, experimentation, and practical deployments with blockchain technology started was logistics [8]. Decentralized applications here reduce time delays, management costs, and human errors. Commonly, supply chains consist of several competing actors. Blockchains provide a framework for coordination, collaboration, and regulation conformance with no central trusted entities. Although these solutions track physical objects (e.g., shipments) and, in some cases, their physical parameters, they focus on object tracking and not on their autonomy. These solutions support efficient and trustworthy planning, scheduling, monitoring, and validation of logistics activities. In [9], the authors pointed out that some of the key benefits of integrating the IoT and blockchains include freight tracking, temperature control, carrier authentication, fast delivery through the continuous monitoring and readjustment of routes, delivery receipts, and payment and vehicle authentication. A survey [10] of the role of blockchain technology in achieving sustainability in manufacturing and product lifecycle management gave an overview of the metrics for adopting blockchains in these domains. They position blockchain solutions as a digital twin for securing manufacturing and lifecycle management data.

Another active field of research prototyping is the Energy Internet. Possible uses of decentralized principles are demonstrated in plug-in electric vehicle charging and peer-to-peer energy trading, combined with distributed energy resource management and green energy. Energy supply and origin certificates are commonly tokenized with distributed ledger technologies to facilitate tradable tokens for novel and flexible trading mechanisms [3].

Use cases related to industry and manufacturing are found in robotics, machine sharing, and manufacturing servitization. Swarm robotics [11] requires efficient coordination to execute mission activities. Blockchains can facilitate this, along with a transparent recording of robots’ actions and improved security. A study of secure coordination mechanisms to identify Byzantine (malicious) members in robot swarms demonstrated [12] the feasibility of the decentralized approach. Performance comparisons of the traditional and blockchain-based approaches indicated a clear advantage of the latter.

Sharing idle or excessive machine capacities [1] optimizes the utilization of the infrastructure, reduces the costs of ownership, and increases the flexibility of manufacturing to adapt to the changed requirements. Two aspects have to be addressed to achieve this. First, transparent process traceability is needed for service configuration, ordering, and keeping track of actual machines performing tasks, applied tools, and process parameters (raw process values and aggregated key performance indicators (KPIs)). Second, a service marketplace must be established, possibly automated, and well-integrated with existing enterprise resource planning and manufacturing information systems. In [1], the authors presented a prototype of a decentralized machine-sharing economy (MSE) approach. A skill and capability model describes the available machine capacities following the DIN 8580:2003–09 standard, which defines the manufacturing process classes of a technical system performing manufacturing process [13]. The marketplace is comprised of several services. The prototype implementation relies on Ethereum-related technology (private network with Parity nodes and Solidity smart contracts). It heavily relies on cloud components, but no clear rationale is given for how the application is split between on-chain and off-chain parts. The authors concluded it was difficult to find suitable and proven blockchain architectures, including design patterns for hybrid DApps and a secure design of smart contracts to facilitate industry-grade solutions. A scalable framework for shared manufacturing proposed in [14] defined a blockchain-based procedure for service execution and considered sharing economy design principles. The authors analyzed the feasibility of the proposed approach and evaluated it in a cross-chain solution.

A blockchain solution for the ice cream supply chain is presented in [15]. This work addresses a real-world use case of machine servitization and trustful competition between refill suppliers. Because of the decentralized approach, the solution can be adapted to small businesses, too. They would otherwise not be competitive customers for big machine manufacturers and refill suppliers. The lack of trust among the participants is reflected in the privacy requirements. These were addressed by using different channels (i.e., similar to a separate subchain) for each triplet of machine client, machine manufacturer, and refill supplier. The solution is implemented on the Hyperledger Fabric blockchain platform and enables the immutable and private identification of devices and objects and traces all refills in a machine. The research also proved that their architecture could be implemented in resource-constrained IoT devices. Raspberry Pi nodes represented the machines in this experiment.

The role of blockchain technologies is also explored in service-level agreement (SLA) management. This research focuses on SLAs in cloud service provisioning, which is an integral part of digitalization in industry and manufacturing. Traditional cloud service management approaches face problems such as poor management and auditing of SLAs, dependence on (untrusted) third-party monitoring, and the dominant position of the service provider compared with the service customer [16]. Blockchain-based approaches can be more efficient than the current SLA management because of increased transparency, automated SLA enforcement and conflict resolution, and decentralized audited decision making [17]. Such an SLA can also more effectively act as a legal binding.

The research endeavors on machine autonomy and servitization with blockchains in manufacturing in this section show that this is a relevant research domain. They demonstrate the role of decentralization in different use cases but rarely focus on and detail the architectural approaches and smart contract designs at a level that is expected in business environments. Smart contract solutions are mostly very simple single-contract solutions that are not scalable or updatable. Data models are frequently not given or use on-chain and off-chain resources inefficiently. Mixed participant identification approaches are used, including combinations of blockchain identities, off-chain certificates, and cloud-based authorization. This effectively prohibits the machine from acting as an economically independent entity.

## 3. Decentralized Applications for the IoT

Machine autonomy and servitization require a mix of technological approaches, and distributed ledger technology is one of these building parts. In [4], the authors listed the novel requirements for the ICT infrastructure, raised by economically autonomous machines engaging in business relationships. These requirements include availability and scalability, reliable and trustful transaction management, identity management for machines and other participants, interoperability, and overall ICT security. DLTs are a valuable contributor to many of these requirements. However, autonomous machines in manufacturing use cases are cyber-physical systems. This means that in a decentralized application built on DLT, we do not only deal with blockchain networks, protocols, and transactions.

Distributed ledger technology-based IoT applications can be seen as a special case of DApps. Specific aspects include DL network performance, smart contract and end-device security (e.g., device access to its BC key stores and accounts), and implementation of the off-chain applications in constrained IoT devices. Additional aspects also reflect the careful selection of the DLT role in the desired decentralized machine autonomy applications. We also have to define the aspects [18] related to the physical manifestation of the designed system, such as securely attaching constrained physical devices to the blockchain network, binding other physical objects (e.g., assembly parts) to the chain, and trusting the data provided from the physical world (with, for example, blockchain oracles). IoT solutions frequently impose industry-grade requirements on the underlying DL networks and their development and support ecosystems.

Running decentralized applications requires a triplet of building blocks: a DLT network, on-chain application logic in smart contracts, and off-chain application parts for the user, machine, and cloud interfaces. Various blockchain technologies and platforms exist, and they can be further implemented in different blockchain networks. A BC platform (e.g., Ethereum) can be implemented in public main (the Ethereum Mainnet) or public test networks (e.g., Ropsten or Rinkeby). Using the same Ethereum node software, we can set up private or consortium blockchain networks. They might share the same or similar blockchain platforms as the public networks but differ in terms of network governance, access, settings, and performance. Smart contracts are sets of programming code executed in a virtual machine (e.g., Ethereum Virtual Machine (EVM)), which is part of the blockchain network. Different programming languages and smart contract approaches exist to assure the finality of smart contract execution (i.e., a common agreement about the execution outcomes). In Ethereum, smart contracts are mostly developed in the Solidity programming language. A smart contract is identified by its unique blockchain address, has a balance, and accepts, processes, and sends transactions. Because they are deployed to and executed in the blockchain network, we refer to them as the on-chain logic. Traditional user, machine, and cloud interfaces are the off-chain logic, which interacts with the smart contracts. Off-chain applications independently perform some blockchain tasks (e.g., create and sign transactions or react upon events emitted by the smart contracts). Using the blockchain nodes’ APIs, they utilize other blockchain services (e.g., block creation and consensus and smart contract execution). End users interact with the DApp through web or mobile user interface applications. Embedded applications in IoT devices or server-side web applications similarly access the blockchain services. Developers can abstract much of the complexity of interacting with blockchain node APIs with off-chain client libraries, which are available for different programming languages (e.g., Web3.js or Web3.py for Ethereum). Client libraries facilitate the integration of DL networks and services with IoT applications. Another useful feature is decentralized name services. For example, the Ethereum Name Service (ENS) [19] resolves user-friendly names into Ethereum addresses. Using the ENS, we can point to a changed smart contract instead of the previous one. The applications referring to an ENS name do not have to be changed.

DLTs rely on advanced cryptographic mechanisms and provide several security features in decentralized applications. However, DLT is not a universal security technology and does not equally contribute to all security aspects [3]. It even opens some additional security risks not exhibited in centralized application architectures. The key security objective of DLT and blockchains is integrity derived from the shared and immutably recorded data. The transparent sharing of the ledger data is the fundament for trust in DLT. Blockchain data are therefore not confidential if specific measures are not taken to ensure as much. The concept of the common ledger also strongly affects data privacy. Users’ personal information is not needed for, as an example, the access and use of blockchain services in public networks. However, the blockchain data can determine the entire transaction history and the account balance. Mechanisms to increase privacy include private and consortium networks, permissioned network access, carefully carving the data stored in the ledger, or applying privacy-enabled zero-knowledge blockchain platforms. Privacy issues may appear even if the transaction data are encrypted. In [15], the authors pointed out the problem of competitive participants having potential business insights into competitors solely from the number of transactions exchanged among different accounts.

Smart contract security imposes specific security challenges to be addressed during the entire DApp life cycle [20]. Smart contract security is, in our opinion, one of the key challenges in meeting the production-grade application requirements for business. On the one hand, most DLT platforms, tools, and smart contract programming languages are still maturing, evolving, and changing their features. Therefore, design flaws might exist in smart contract languages and shared libraries, inappropriate SC architecture designs may be taken, or errors and bugs in the SC programming code can be made. The Smart Contract Weakness Classification and Test Cases Registry (SWC) [21] is a comprehensive list of key security flaws in the Solidity smart contract code. However, the immutable nature of the already-deployed smart contract code prevents the efficient updating and upgrading of the SC code if the SC architecture does not carefully consider these options. Some of the vulnerabilities easily mitigated in traditional software systems can therefore present severe risks in decentralized applications. Passive security measures involve smart contract architectures, software engineering techniques specific to the smart contract environment, and code reviews and verification. Only recently, smart contract design patterns, software engineering techniques [22], verification tools (e.g., MythX [23]), and security libraries (e.g., OpenZeppelin [24]) started appearing for the leading SC platforms and SC languages. Active smart contract security measures occur during smart contract execution and refer to smart contract and method access control, active monitoring of the incoming smart contract transactions, and authorizing their actions [3].

Some of the initial expectations [2] about blockchain technology for the IoT included seeking an alternative for centralized cloud-based IoT data storage and management. Decentralized approaches were expected to provide performance and scalability in the cloud backend. A blockchain system can be described as a form of a distributed and decentralized database. However, the high distribution of nodes in the network is not meant to assure performance or bulk data storage, but rather the distributed and decentralized consensus, machine autonomy, and monetary aspects in machine communications [25]. The transactions in BC systems usually have a predefined structure and sometimes an option to include arbitrary data (e.g., a JSON string in an Ethereum transaction). These data were primarily meant to carry the parameter values when calling the smart contract methods. The amount of these data is practically limited by the transaction processing costs and block size, among other factors. Therefore, most blockchain-based IoT data management approaches, such as the IPFS, FairAccess, ENIGMA, or BigchainDB, take a hybrid approach, where bulk data are stored off-chain and the blockchain is utilized for management. The hybrid approach offloads the bulk data from the chain, preventing excessive growth of the chain data, and assures consistency, trust, and decentralized data access control. Hybrid approaches are not limited to the aforementioned decentralized storage approaches but can also include cloud-based storage. The actual architecture of a hybrid decentralized cloud data depository in a DApp is a fundamental step when we define the DApp architecture.

Blockchain accounts and corresponding addresses can be successfully utilized to identify the participating devices in an IoT DApp. Unique addresses are a prerequisite for autonomous participation in blockchain transaction exchanges. If a device in the solution possesses a blockchain address, this address can also identify it. In the enrollment process, the logical identification of a device can be matched to the physical address (i.e., the IMEI number or MAC) or some other non-blockchain identifier (e.g., QR code). Blockchain-based identifications can be used for authorization and access control in smart contracts [20] or off-chain applications [26]. Blockchain-based solutions are even considered (https://login.xyz, accessed on 14 December 2021) in sign-in mechanisms for easy implementation in web-based services, either independently or in an OAuth-compatible manner.

Decentralized applications based on DLT enable monetization in the IoT [20] as a part of the decentralized business logic implemented in smart contracts. The most recognized use of blockchain technology is cryptocurrencies, where the exchange of monetary value between accounts is the primary intention. In IoT systems, some form of financial compensation is sought for the investments and operation of IoT and blockchain platforms and for monetizing data and related contextual information [18]. Similar technology enables payments controlled by smart contracts in the servitization of end services. Economically autonomous devices thus become service providers and consumers and can negotiate, pay, and get paid for the provided services. The addition of monetization is a strong enabler for innovative business cases. Smart contract logic does not have to include monetarization, but it can if needed.

Business logic and payment methods rely on technologically separate platforms and solutions in traditional systems. Public cryptocurrencies require public blockchain networks. These networks might not be equally appropriate for IoT DApps due to public network performance, privacy, and scalability constraints. However, tokenization is an alternative mechanism for value exchange in private or consortium networks. Participants are usually not anonymous in business applications and are granted permissioned access to the network and applications. Aside from the logic implemented in smart contracts, they can also be bound with some legal agreement that defines the value and lifecycle of the tokens in the system. Finally, the emerging cross-chain solutions (e.g., COSMOS [27] and Polkadot [28]) provide the means to combine various blockchain networks in the same decentralized application. With cross-chain solutions, a consortium network for performant and private IoT management could be connected to a public network to enable the monetary aspect of the application.

While the Internet of Things presents a well-established, proven, and industry-grade technological concept, distributed ledger technologies—with blockchains as the leading example—are still maturing. Therefore, the productization of DApps is a specific challenge [29]. Adopters are faced with the rapid development of new decentralized concepts and platforms. At the same time, they need to select and base their research on a relevant blockchain ecosystem which assures them long-term and sustainable solutions and partnerships. Other aspects of the ecosystem are different from the technological platform characteristics (performance, governance, and scalability). These aspects include the available libraries and tools facilitating the development and testing, monitoring networks, validating the solutions, quality documentation, and efficient formal and non-formal support through the community gathered in the ecosystem. An indicator for the decisions about the DLT platform is also a clear roadmap, an open, collaborative development culture and clearly defined mechanisms for cooperation with academia and enterprises, and the scope of prominent use cases. 

Blockchain-as-a-Service (BaaS) providers facilitate efficient blockchain network node deployment, system monitoring, smart contract analysis or testing, and access control. With BaaS [30], DApp developers can focus on application development and use instead of system provisioning. Most of the key players in cloud service provisioning (e.g., Amazon, Microsoft, Alibaba, and IBM) provide some form of BaaS. Two DLT technologies are dominant in BaaS: Hyperledger Fabric and Quorum (Ethereum-based). This prevalence is not surprising, since both are predominantly meant for private or consortium-based blockchain networks utilized in business BC applications.

## 4. Use Case Definition for 3D Printing Servitization

We designed and developed a decentralized application composed of a smart contract platform, off-chain user and machine interfaces, and an underlying consortium proof-of-authority blockchain network. In our approach, we applied blockchain technology where it shines. We did not impose the expectations it struggles to meet, especially when scaling out the solution and advancing it toward system prototype demonstrations in operational environments. We first defined the actors and corresponding use cases. The research in [1], for example, defines three application actors in their machine-sharing economy example on process traceability in distributed manufacturing: client, owner, and operator. We added two additional actors. First is the solution provider, who is crucial for the productization of the solution and the security expected in business environments. However, this does not mean adding another entity in the use case but shaping the design of the smart contract platform. Second, we introduced the machine as an autonomous actor. A machine does have a proprietor who registers a machine in the system and claims its earnings. Apart from that, machines autonomously participate in all business arrangements carried out among the actors in the decentralized application. All the actors in the system are identified and authorized to execute smart contract methods by their blockchain accounts. Unlike some other related approaches [1] which rely on cloud services for basic system features (e.g., user authorization), the entire trust in the system and the business logic are encoded in the on-chain smart contract platform.

The expenses of blockchain network infrastructure provisioning are compensated by each consortium member running nodes. The consortium network imposes no transaction costs during the operation. Tokens with a fixed value are used for cost clearing in machine service provision and consumption. Currently, the tokens act as vouchers, where the value is fixed in FIAT and determined with a business agreement in the consortium. A cross-chain solution would assure immediate compensations with public cryptocurrencies, too.

The solution utilizes a hybrid data storage approach that integrates on-chain and cloud storage for trust and efficiency. The off-chain cloud storage is an add-on integrated into the on-chain logic. Apart from flexibility and upgradeability, which are not self-evident in decentralized applications, this builds the foundation for even the most futuristic sharing economy scenarios in manufacturing with economically autonomous machines. We concretized the machine sharing principle in a 3D printing use case. We defined a data model that was secure, efficient, and general enough to accommodate the selected use case during the design and development. At the same time, it could directly support or be easily adapted to other machine sharing use cases, such as collaborative robotics.

In the continuation, we introduce the service workflow and the actors in more detail. We present the smart contract platform as well as the data and event models. Finally, we present the user and machine interfaces of the decentralized application.

### 4.1. Actors

**Figure 1:**
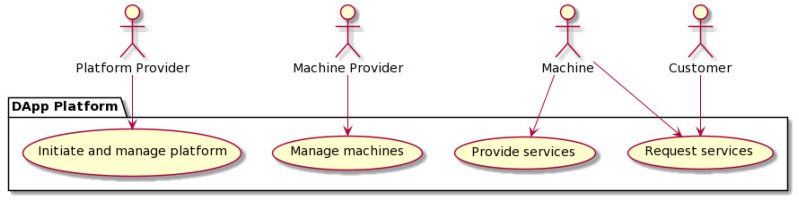
Use case diagram of actors and activities.

Four different actor types are foreseen in our solution: platform providers, machine providers, machines, and customers, as can be seen in Figure 1. These can be people, devices, or legal entities in the real world. To participate in the decentralized solution, they need a blockchain account and are identified by a blockchain account. The business objectives of a particular actor type are reflected in the role-based access control in the smart contract design. The mapping between the actor type and their effective roles are given in Table 1 and explained in more detail in Section 5.1.

A platform provider is the initiator of the solution that deploys the initial smart contracts and applies the initial settings of the system. The initial settings comprise the mappings between the deployed (multi) contracts and assigning roles to blockchain accounts. The provider is also responsible for the management of the platform during operation. The management includes smart contract updates and registration of the machine provider addresses. Apart from that, it is not involved in any other business logic. There is only one participant of this type in the system, and it will usually be the system integrator of decentralized solutions.

Machine providers are owners of manufacturing machines, which they would like to share through the decentralized platform. Usually, they would represent a legal or business entity dedicated to a manufacturing business. Machine providers perform the basic management of the machines, including registration of new machines, configuration, updating a machine’s status and information, and removing them from the system. There can be many machine providers in the system. The more there are, the richer the servitization offering enabled by the system.

Customers can place and monitor a service order but not accept or execute one. This actor presents a service consumer or a company needing manufacturing services from the system.

Machines can be any manufacturing devices, including collaborative, welding, additive or assembly robots, 3D printers, grinding, milling, mold injection, or CNC machines. Our use case was elaborated in more detail for 3D printers. A machine can act as a service provider or a service consumer. It includes all the functionality of a customer actor and additional functionality for service provisioning. It can place a service order or accept and execute one and is highly autonomous. A blockchain account identifies a machine, and it can hold funds, negotiate for appropriate bidding, be compensated for the provided services, or be penalized for faulty executions. In our case, the machines still have an owner who can retrieve the machines’ funds or deregister a machine from the system. The existence of a machine owner reflects the current and near future of machine autonomy, where machines still are not independent legal entities. However, our solution could enable economically autonomous machines if the legal frameworks allowed for it.

### 4.2. 3D Printing Servitization Workflow

When a customer or a service-requesting machine starts placing a new service order, the system suggests an appropriate service-executing machine. The smart contract platform manages a list of currently vacant machines of the required type.

**Figure 2:**
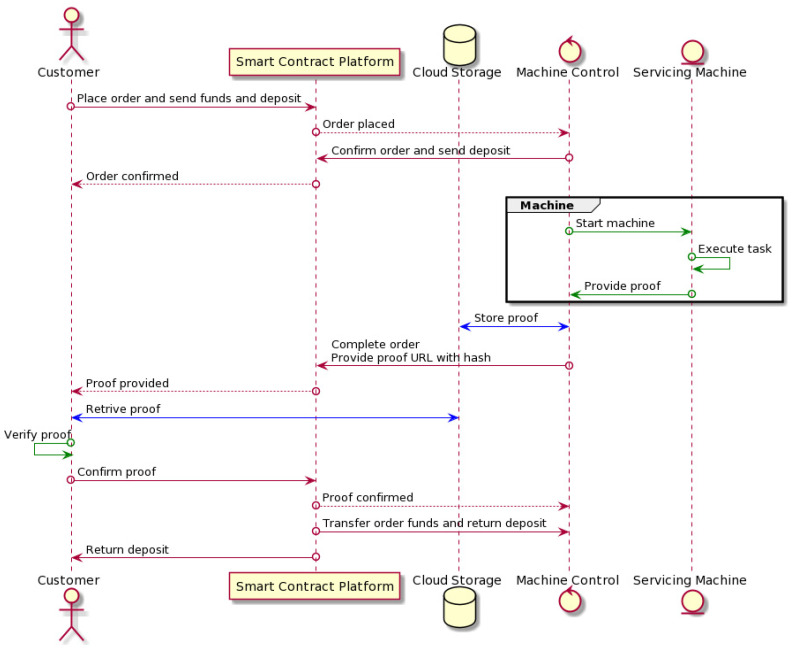
Order processing sequence chart.

The 3D printing service order processing is depicted in Figure 2. A customer places an order with a transaction sent to the smart contract platform. The transaction, which includes the address of the selected machine, specifies the order details and includes funds for the service payment and the agreed deposit sum. For 3D printing servitization, the order specification includes the expected deadline and the URL pointing to the 3D printing specification in an STL file. Deposits in our system guarantee that the requesting and providing parties are committed to the service arrangement.

The service-provisioning machine receives the order placement notification, verifies the STL model file, toggles its status to busy, and confirms the order. Before the order confirmation, the customer can cancel the order. If the machine rejects the order during this negotiation (e.g., because the machine status no longer allows for the timely task execution), the deposit is returned to the customer. In the order confirmation transaction, the machine also includes the deposit as the guarantee. The platform is an escrow, which secures the funds and deposits of the requesting and provisioning parties during order processing. In Figure 2, we separate the machine control from the actual 3D printer for clarity. The following action thus occurs internally in the machine. The control unit starts the 3D printing procedure. When printing is completed, the printer notifies the control about it. At the same time, the control system records a video of the printing process and stores it in the cloud server. This record and the successful printer status are the proof of the task’s completion. Upon completion, the machine sends a notification to the blockchain that the task has been executed. The notification transaction also records the proof URL and file hash to the blockchain.

Once the task is completed and the proof provided, the customer can retrieve, verify, and confirm the proof. Then, the platform notifies the servicing machine about the confirmation and transfers the service funds and the deposit to the machine’s address. Similarly, the security deposit is returned to the customer’s account.

This use case is focused on 3D printing, which allows for certain simplifications. For example, selecting an appropriate manufacturing machine is straightforward and does not require an elaborated decision mechanism. In the current implementation, a round-robin principle is used for machine selection. The selection procedure can be easily adapted to a more sophisticated selection procedure that involves customer selections and confirmations and considers criteria such as the reputation of the machine, detailed KPI or raw process values, or an elaborated execution schedule. The advanced selection would take place off-chain but would be based on data recorded in the chain. The use case and the entire solution can be easily adapted to other related use cases, such as collaborative robotics or manufacturing.

Order negotiations are also rather basic now in this use case. We have already experimented with auction modules in the smart contract platform to introduce advanced machine-to-machine biddings and business negotiations about orders.

Initial platform deployment, set-up, and management workflows, including updates and upgrades, are a part of regular DevOps procedures and system administration and are not use case-specific. We explained these procedures in [20]. In Section 5.1, we explain how they are related to the security aspects of the smart contract platform. In Section 5.2, we explain the rationales behind the hybrid blockchain and cloud data storage. Details of the customer and machine interface implementations are given in Section 5.3.

## 5. Solution Design and Development

The proposed solution is a comprehensive decentralized application comprised of a smart contract backend, off-chain machine and user interfaces, cloud-based storage, and an underlying consortium-based blockchain network. Every part of the DApp was carefully designed. We paid special attention to the data and event models.

We selected the Ethereum ecosystem for implementation and practical experimentation based on its characteristics and our previous good experience with Ethereum technology. Ethereum provides a proven technical background for DApps, systematic technology development, extensive examples, libraries, integrated development environments, security validation tools, and documentation. It has a large developer community and a clearly outlined roadmap toward Ethereum 2.0. The Ethereum virtual machine and Ethereum smart contracts are also used in several other distributed ledger ecosystems, so a relevant part of our solution can be directly replicated there. Another benefit of Ethereum is its application in public as well as consortium networks and the possibilities for cross-chain integrations. Finally, we anticipate that Ethereum-based blockchain network provisioning for business environments will soon be even more widely available than BaaS. Ethereum BaaS will enable focusing on application development and provisioning instead of setting the nodes and a reliable network.

### 5.1. Smart Contract Platform

Our previous research proposed a modular multi-smart contract platform which was upgradable and updatable. The platform comprised service-agnostic, service-specific, and auxiliary smart contracts [20]. This underlying smart contract platform facilitates several security contributions essential for the productization of decentralized applications. In each smart contract module, methods are provided to freeze and update a smart contract if a security vulnerability is found or functional extensions are required. This facilitates the efficient management of security updates and upgrades. Smart contract tunnels (SCTs) ensure access control mechanisms at the level of message senders. SCTs interconnect only the valid SCs in the platform and prevent malicious SCs from accessing the platform methods. At the level of transaction origin, we provide additional per participant role-based access control to the platform methods. Each participant can be granted one of four permission roles to control the access to service-specific and platform management smart contracts and methods. We adapted this multi-tenant platform for the decentralized machine autonomy use case.

SmartContractIndex and SmartContractAdministration are key platform management contracts, and they remained in their roles. The Core module only served as a relay smart contract; it implemented no service-specific logic (compared with the ChargingStationCore in [20]). It was the single access point for any method calls from the off-chain applications to the smart contract platform. The Orders contract implemented the business logic. This contract was service-specific and reflected the business workflow and order processing. It also provided escrow for service providers and consumers during the order execution and held the service payment until the order was completed. For the operation, it relied on data in the MachineDirectory. MachineDirectory is a service-specific on-chain registry of the machines utilized in the solution. Every machine is registered with its blockchain address, type, state, and machine info (JSON structure with detailed machine description) as well as the address of the machine owner. MachineDirectory entries can be created and updated only by machine owners. The Orders contract may change only the machine’s state to reflect the current workflow execution status. MachineDirectory has rather simple functionality, but it was excluded from the Orders so that it did not have to be modified for another servitization use case.

**Figure 3:**
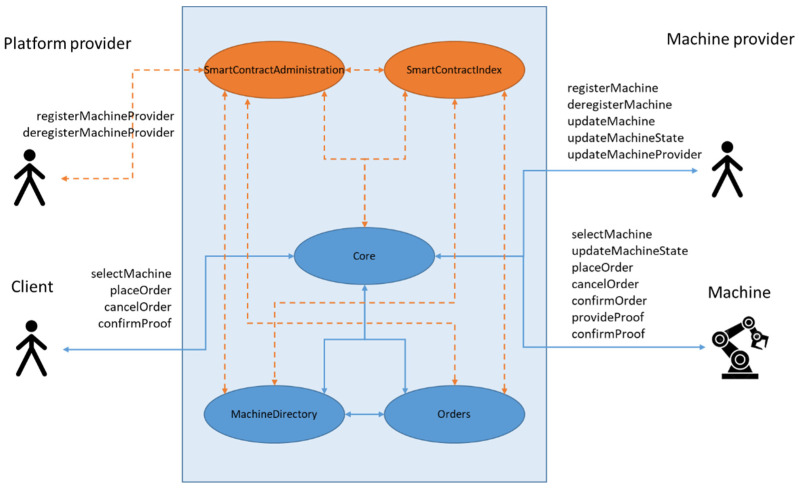
Top-level smart contract layout and actors.

Figure 3 depicts the top-level architecture of the smart contract platform. The machine provider, machine, and customer actors only access platform functions through the Core module, which redirects the calls to corresponding service-specific contracts while ensuring that the method access policy matches the actor’s role.

SmartContractIndex is a registry of all smart contracts in the platform. An index points to the currently valid contract version and holds the addresses of the deprecated versions. SmartContractIndex also holds current subscriptions in place between the contracts on the platform. In this way, it defines the endpoints of the smart contract tunnels. Aside from its registry function, SmartContractIndex notifies all the subscribed contracts about the index changes. It thus assures that any update is seamlessly propagated through the platform.

SmartContractAdministration provides common role-based access control in the smart contract platform. It registers all the participants and their possible access roles of owners, admins, or registered users. This way provides all the other smart contracts with corresponding access rules in inter-contract calls. 

The actor types need to be distinguished from their smart contract access roles. The effective mapping is given in Table 1. While the participants are real-world entities, roles are attached to their blockchain accounts:

- Owner is a role derived from Ethereum smart contract principles and refers to the account the contract was deployed from. The owner of a smart contract cannot be changed;
- Admins are responsible for day-to-day configuration, such as registering new users in the smart contracts. One or more accounts can be assigned to the Admin role. We recommend that a platform provider uses a separate blockchain account for security reasons for the Owner and Admin roles. The Owner account is responsible for the initial platform deployment, critical migrations such as smart contract updates and upgrades, and appointing new Admin accounts to smart contracts;
- Registered users are machines and machine providers. This means that an account in the decentralized system can only execute functions related to these two roles if registered with the corresponding role in the SmartContractAdministration;
- Any account in the system can invoke customer functions, as the customer actor is an Unregistered user. However, the use is still limited by the business logic (e.g., machine state or sufficient funds for service compensation or an invalid machine state). If, for example, the solution was extended to include the loyalty mechanism for customers, the role of the customers would be changed from the Unregistered user to the Registered user to increase the security to the level expected by such functionality.

If needed, additional service-specific modules could be added to the platform. For example, a Loyalty module could ensure an ERC20-based loyalty system in the form of a non-fungible token or a stable coin. Similarly, a Parachain module would attach the presented solution to a relay chain and link it to other blockchain systems. In this way, the solution could combine the performance and privacy of a consortium blockchain with the monetary and DeFi aspects of a public cryptocurrency network. In this case, the escrow function would become more complex and most likely retrieved from the order-processing module to a separate one, too. An Accumulator module would enable the registered users to retrieve their funds from the Escrow smart contract.

Ethereum smart contract best practices recommend a relay smart contract [22] to point to the latest version of a contract, enable upgrades and updates, and act as a single point of interaction in a multi-contract platform. Our Core contract does this. There are alternative approaches to assure similar functionality, but none fulfill all the needs of our solution. ENS enables mapping between a permanent identifier and a changeable smart contract address. However, it does not support any programmable logic, such as push notifications to propagate information about address changes proactively. Another approach to upgradeable modular contracts is the “diamond cut,” proposed as Ethereum Improvement Proposal (EIP) 2535 [31]. This draft document proposes a proxy contract that supports using multiple logic contracts and facets that supply one or more than one smart contract function. Events are emitted upon changes in diamond functions. Unfortunately, the diamond approach facilitates no security functions, such as SCT or role-based access. The OpenZepplin Upgrades smart contract security library enables a proxy upgrade pattern [32] that can be controlled by any type of governance. The problem with this pattern is that it is meant primarily for a single contract solution. In our case, this approach would require five separate proxies, with one for each module in the platform. With the extension of the functionality and adding additional modules (e.g., Loyalty and Escrow), management of the replicated proxies would become challenging.

### 5.2. Data and Event Model

A data model is important for efficient and secure hybrid on-chain and off-chain storage. The event model serves as a notification system between the smart contracts and the off-chain applications, and importantly, it increases the responsiveness and usability of the user and machine interfaces. 

#### 5.2.1. Data Model

The data model defines the three levels of variables, parameters, and data sources needed in decentralized applications. The data are stored on-chain and off-chain and thus present a hybrid DApp architecture. The model design considers the expectations of trustworthy data recording, flexibility to adapt the solution without unnecessarily affecting its availability, and the limitations of blockchains when storing large amounts of data. Therefore, we utilized the smart contract variables and arbitrary JSON data structures stored in the chain and off-chain cloud data storage for large binary objects.

Solidity provides several elementary variable types. If a new variable has to be added to an existing smart contract or the variable type changed, this would require a new smart contract deployment alternative to the previous version. Although upgrading is possible in our smart contract platform, we carefully selected the set of smart contract variables and limited it to the essential ones. This limitation minimized the need for smart contract modifications, even if some business logic modifications were applied (e.g., adding new machine types or changing a description of a machine provider). Any extensive or non-essential upgrades may jeopardize platform security and availability. In this way, we kept the platform rather generic, but at the same time, we did not prevent functional extensions and business adaptations. All smart contract values were read-only and could only be modified through modifier functions, where RBAC was applied for security reasons.

**Figure 4:**
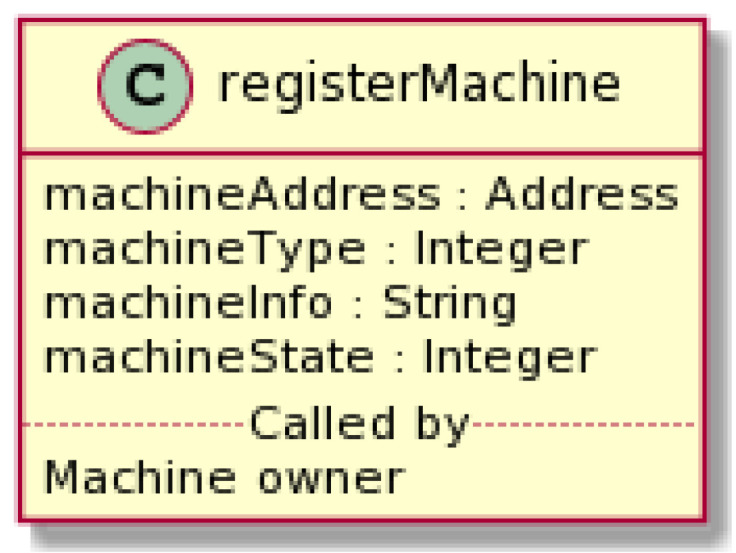
An example of a smart contract function and corresponding parameters.

Figure 4 gives an example of the input parameters with their corresponding value types in a registerMachine function. The machineAddress parameter is a type address and can only hold Ethereum blockchain account addresses. The machineType and machineState parameters are type integers. There is no other variable to describe the variety of possible machine types or states in the smart contract platform. Instead, we used enumerators to interpret the actual meanings of the machineType and machineState variables. If a new machine type were to be added to the system, we would just choose a new enumerator for it.

To maintain the high flexibility of the solution while benefiting from the trusted ledger records, we kept other values in the JSON strings. These values are not required for the on-chain logic but are essential for the meaningful operation of the user and machine interfaces. For example, detailed information about the machine is given in the machineInfo parameter, which is a JSON string. Smart contracts do not impose any limitations on this JSON structure. It can therefore be used to keep values that might be arbitrary and is easily extended with additional values, such as an additional machine description, location, and clarification of its properties, version, or model. These values are duly recorded in the blockchain but not directly queried by smart contracts. Instead, JSON strings are parsed, and the values are used in off-chain application parts.

Apart from the smart contract values and JSON data kept in the chain, the solution requires digital content that is too extensive or dynamic for efficient on-chain storage. For example, this is a 3D printing specification in an STL file that describes the three-dimensional surface of the object to be printed. The file can be in ASCII or binary representations. Similarly, we record a video of the entire printing process. As described in Section 4.2, this is used as proof that the order was completed. The video is automatically stored on a cloud server. To combine the cloud resources that are part of the business process (as inputs or outputs) in a hybrid solution with the blockchain data model, we permanently store JSON structures with hash values, URLs, and metadata in the chain. Although not available in the chain, the authenticity of the off-chain resources can always be transparently verified with the accompanying chain data.

#### 5.2.2. Event Model

Solidity events give an abstraction on top of the EVM’s logging functionality. Applications can subscribe to these events through the RPC interface of an Ethereum client. Events are inheritable members of contracts. When we call them, they cause the arguments to be stored in the transaction’s log. These logs are associated with the contract’s address and are incorporated into the blockchain [33]. In our solution, the events reflect all the key changes in the workflow (e.g., an updated state of an order or machine, registration of a new machine, or changed information about a machine owner). They invoke callback functions in the user and machine interfaces while listening for these events.

In the example given in Figure 4, the registerMachine method accepts the input parameters upon the invocation. It executes the logic (i.e., registers a new machine in the machine directory) and emits the machineRegistered event. Similarly, several other event types are emitted during the execution of the order-related functions. These events give notification regarding the updated order state. The user interface application (see Section 5.4) sets an orderUpdate event filter in the blockchain access node JSON-RPC API to receive this notification. An API call registers the event listener with the appropriate filter setting. The notifications are pushed through the WebSocket connection from the node to the off-chain application. In this way, the user interface immediately changes (e.g., the order state label in the management web page).

The machine interface also needs to listen to the events about the order processing. For example, when a customer or a machine places an order, it calls the placeOrder function in the Orders smart contract. This function checks the message value, which has to equal the value of the order and the obligatory order deposit. Upon completion, the function emits an order update event with the status set to created. The target machine has an event listener set that catches this event and continues with the order processing (i.e., it verifies the input STL model file and confirms or cancels the order).

Table 2 gives an overview of the key events emitted by the smart contracts in the platform, the indication if they are intercepted by the user or machine interfaces, and the consequent effect of the event in the off-chain application. Events are not relevant only for the immediate off-chain application notifications. We can also access the past events and their timestamps. In this way, we can reproduce and visualize the entire order processing history. The settings that send transactions to the chain and are made from the machine or user interfaces do not necessarily require the consequent event notifications. There is always a transaction confirmation that can be utilized for the interface status update.

### 5.3. Customer and Machine Interfaces

With the customer interface, we place an order. The customer provides a URL to the STL model file with the printed object description and the deadline for the order’s completion. This is included in the order transaction and recorded in the blockchain together with the file hash for verification. If needed, the customer or servicing machine can cancel the order.

Machines—in our case, 3D printers—interface the blockchain backend using embedded software on a single-board computer. The machine interface application is Python-based and uses the Web3.py library. It interacts with the physical device via a USB interface and the blockchain via a JSON-RPC API at the blockchain node over the IP network. The interface on the physical device enables complete control of the printing process.

Potential challenges for future machine autonomy are the device constraints and the hardware security. IoT devices may be limited in their computation and communication capabilities. However, this challenge is more exposed in non-industrial use cases (e.g., environmental sensor monitoring). We can usually count on a sufficient power supply and controlled connectivity in industrial applications. This minimizes the communication constraints. We can also ensure enough computation capabilities with gateway devices to execute blockchain-specific operations in the device. Hardware security refers to trusted storage and access to the blockchain accounts that identify the device in the system. With economically autonomous devices, these accounts can hold monetary value, too. The current machine interface functionality could be integrated into the device’s firmware instead of relying on a gateway device. The hardware could provide a trusted execution environment (TEE) to maximize hardware security.

### 5.4. Management and Monitoring User Interfaces

We created a web-based user interface for efficient management and monitoring of the system at the business process level. The entire process is indeed recorded in the transactions. The chain blocks and the chain data can always be analyzed with network scanning tools, such as Etherscan for public Ethereum networks or Hyperledger Explorer for private networks. We can view or query blocks, transactions, or account details and monitor the network’s operation with these tools. However, from the business perspective, we expect more user-friendly, high-level insight that is matched with the business process. The management UI runs in a blockchain-enabled web browser so that a user can be identified by a valid blockchain account, which is registered in the SmartContractAdministration module. Blockchain-based authentication in the browser also provides control access to the management and monitoring functions. Based on the actor type, parts of the interface are enabled or disabled. The machine providers can register and manage their machines or update the machine’s provider data in the interface. An administrator can appoint new machine providers. Both actors also have detailed insight into the order execution. They can filter the order list by address, status, and timestamp.

**Figure 5:**
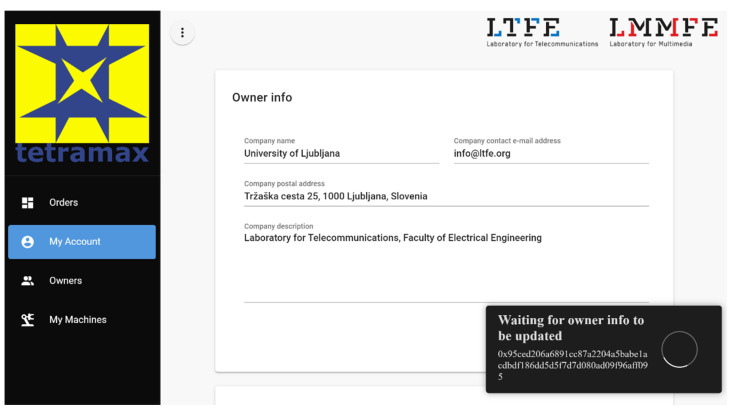
Web management and monitoring user interface.

Figure 5 shows a snapshot of the management and monitoring user interface. The current user of the management interface is registered as a machine provider and administrator. They can edit and update the machine provider’s details. The user interface indicates the pending transaction (bottom right) for these changes to be committed. As machine providers, they can list their machines and update the machine’s details.

The interface utilizes the proven Web3.js client library [34] for interacting with the blockchain node API. The UI application logic is written in JavaScript, and web pages are created using well-known web technologies.

### 5.5. Consortium Blockchain Network

We established a consortium proof-of-authority (PoA) blockchain network to meet our solution’s security and performance needs. PoA is a consensus mechanism which successfully replaces the traditional proof-of-work (PoW) mechanism. While PoW has, to some extent, a meaningful role in public blockchain networks, it is extremely energy-consuming and has significant jitter in block creation times due to the randomness in the consensus. Public PoW blockchain networks comprise many mining nodes, which increases the network propagation times and limits the performance in terms of transactions per second. The implemented system for our use case is not public. It is a system provided by a known platform provider to known machine providers. If needed, their business arrangements reflected in the platform can be bound by legal agreements. Therefore, there is no need for a public network. The PoA mechanism was created for consortium-based blockchain networks. Only a set of known trusted entities is allowed to set up confirmation nodes and thus participate in the block creation process. The mining process does not depend on extensive computation for random calculations of new block hash values. PoA is therefore energy-efficient and supports arbitrary block times and sizes, increasing network performance and decreasing transaction latency. The restricted network access, which can be limited only to registered participants, improves the system’s privacy.

An important feature of PoA which is especially valuable for machine autonomy use cases is a free transaction environment. This means that the gas price is set to zero, and the blockchain network imposes no transaction costs. The gas is still calculated for each transaction and remains relevant for combining the pending transactions in a new block. Another benefit of free transactions is that the native cryptocurrency can now be easily used for manufacturing service payments. The platform provider manages the distribution of the PoA Ether among the solution participants. The currency can serve as a voucher (namely a token without ERC smart contracts), and its value can be mapped to FIAT at a fixed price.

The key network configurations are set up in the genesis file. It sets, for example, the block time and block size, allocates funds to the initial blockchain accounts, and specifies the boot nodes. We set the block size to 8,000,000 gas and the block time to 3 s.

We used three node types in the system: boot, confirmation, and access nodes. They all used the same Ethereum blockchain node software and only differed in their functions in the network. One node instance could perform all three functions, but we preferred to keep them separate entities for increased node and network robustness.

The boot nodes are index nodes in the network. At the network’s genesis, they point to the initial set of confirmation nodes and later to the nodes added to the network afterward. A newly added node points at a boot node during its initialization. This ensures rapid synchronization with the network for the flawless execution of blockchain network communications. The confirmation (i.e., mining) nodes implement the consensus mechanism. A new confirmation node can only be initialized in the network with a copy of the initial genesis file. The access nodes do not have to participate in block creation. They expose APIs for off-chain applications to interact with the blockchain network and to access the services provided by the network. In our experimentation, the need for separate access nodes became evident because the access nodes appeared to be resource-consuming. The resource consumption was correlated with the number of API calls and, in particular, with the number of transactions created. The boot and confirmation nodes did not exhibit these changes in resource demand.

**Figure 6:**
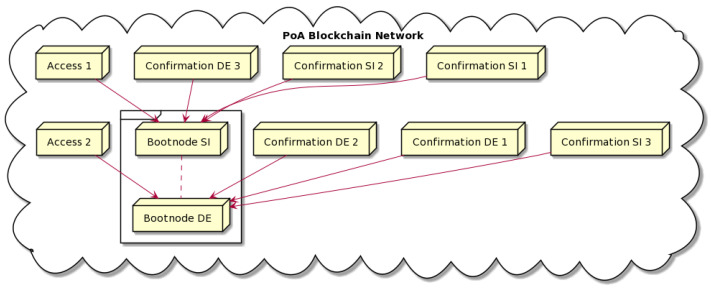
PoA network architecture.

Figure 6 shows the architecture of the blockchain network. The nodes belong to two consortium partners. Some were located on-premises in two different countries, and additional ones were installed in the cloud. The initial boot node was installed on-premises at the first consortium partner. Later, several confirmation nodes were added at both consortium partners along with some additional ones at a cloud hosting provider. Each partner had one access node for the off-chain applications.

Because the number of nodes in the network was low compared with a public network, and we only added new nodes if agreed upon by the consortium, we could apply IP network access limitations (firewalls) to protect the network from unwanted external access. Setting up proprietary nodes was the only viable solution that allowed for flexibility and full control of the network during our testing. With the emerging BaaS providers, a viable alternative would be a hosted blockchain network compatible with Ethereum technology.

## 6. Evaluation

**Figure 7:**
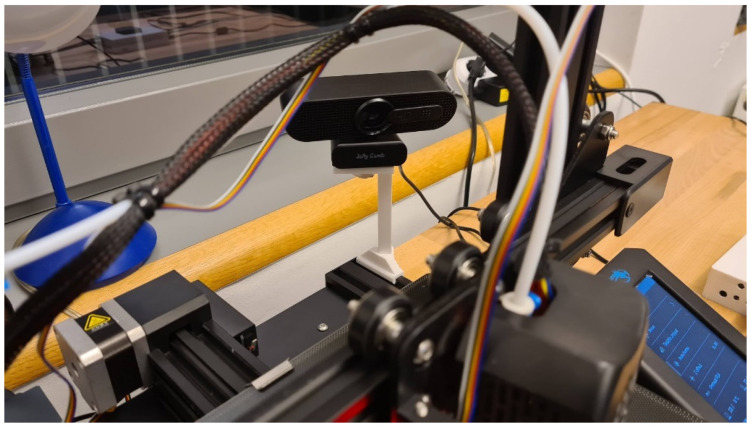
3D printing set-up and camera for process recording.

Figure 7 shows a detailed image of the actual experiment set-up (i.e., the 3D printer with a camera mounted to record the manufacturing process). We tested the operation of the developed system, and it provided the required functionality successfully. A successful order execution resulted in four transactions for order placement, confirmation, task execution, and provisioning of the proof. In addition, the servicing machine emitted two additional transactions for machine state updates. Canceled or rejected orders resulted in only two transactions. Each addition or information update resulted in one transaction in the management and monitoring user interface.

It was impossible to estimate the exact average transaction rate per unit of time, because the actual manufacturing process could last from a couple of minutes to a couple of hours. For example, if the printing task took 60 min, this would result in a transaction load of 0.00167 Tx/s. In other words, the system could run several hundreds or even thousands of such orders simultaneously to reach an average transaction load in the order of magnitude of 1 Tx/s. These are the transaction loads that are easily accommodated by the underlying network. We analyzed the transaction sizes in the typical order execution and found 200,000–400,000 gas transaction sizes. The actual figures might vary because of different sizes of input parameters, but these variations were small and kept the size estimate in the given range. We estimate that with the current block size of 8,000,000 gas and a block time of 3 s, our PoA network was capable of about 20–40 Tx/block or 7–13 Tx/s.

In addition, we carried out some network performance evaluations. We loaded the network with randomly generated transactions sent to the smart contract platform, with average transaction rates of 1, 2, 3, 5, and 10 Tx/s. We monitored the transaction pool at the confirmation nodes. The network was able to accommodate all these transaction rates. Interestingly, the bottleneck proved to be the transaction creation process, which required the composition of the appropriate data structure and digital signatures and hashing. While the system resource of the confirmation and boot nodes remained unaffected by the increased transaction rates, the node which was emulating the machines and customer interfaces showed increased CPU usage. As the transaction rates applied in the network evaluation were substantially higher than the ones we would realistically expect in practical solution deployment, we did not further investigate the reasons for the increased CPU usage. At the same time, this was not a blockchain network issue, but rather a problem of machine interfaces if they were resource-constrained. However, the machine interfaces could be implemented more efficiently without using Web3 libraries or could even create the transaction from scratch to reduce the resource consumption during transaction creation.

## 7. Conclusions

We presented the design, development, and implementation of a hybrid decentralized application for servitization in manufacturing environments. It assures machine autonomy, such as for 3D printing in our case. Machines have their own blockchain identities and can place, negotiate, execute, and charge for manufacturing services. The solution can be used or easily adapted to other manufacturing domains.

We carefully addressed two critical success factors for integrating blockchains and the IoT in manufacturing and industrial environments: the security of the smart contract platform and the data and event models. These two aspects become especially relevant when progressing the solutions from laboratory validation to demonstrations in relevant or operational environments. The smart contract platform is upgradable and updatable, and it uses a proxy contract to control the access of smart contracts and role-based access control for function calls for blockchain users. The approaches to the security design of the applied smart contract platform are now comparable to those in traditional software engineering. Data and event models are fundamental for the hybrid data storage, DApp architecture, and responsiveness of off-chain interfaces. We deployed and evaluated the DApp to a consortium blockchain network for performance and privacy, which proved to be performant enough to support the selected use case. The performance requirements might differ in different use cases and need to be estimated separately.

Our research shows that with the proposed data and event model, we could efficiently separate on- and off-chain data and assure scalability of the DApp without compromising the trust. We demonstrated that the secure upgradeable smart contract platform, which was adapted for machine servitization, supported the business workflow and, at the same time, assured common identification and authorization of all the participants in the system, including people, devices, and legal entities.

Different use cases could be reflected in the extended smart contract platform. Additional escrow, bidding, or machine profiling and selection modules can be added to the platform to separate their on-chain functionality from the existing ones. Still, the data and event model, as well as the smart contract security and access control features, would remain. Similarly, a parachain module would attach the presented solution to a relay chain and link it to other blockchain systems to facilitate a consortium network for the performance and privacy of the IoT and a public cryptocurrency network for monetary aspects of the business solution. Our solution thus facilitates the integration of decentralized approaches with traditional ICT systems in terms of hybrid application and storage architectures and the security standards for smart contract design.

## Figures and Tables

**Table 1:** System participants and their foreseen smart contract roles.

Participant or Role	Owner	Admin	Registered User	Unregistered User
Platform provider	Yes	Yes		
Machine provider			Yes	
Machine			Yes	
Customer				Yes

**Table 2:** System events and interfaces.

Event	UI	MI	Immediate Effect
orderUpdate	x	x	The user interface retrieves and displays an updated status of the order. Machine interface
machineOwnerRegistered	x		The user interface retrieves and displays an updated list of machine owners.
machineOwnerUpdated	x		The user interface retrieves and displays updated details of a machine owner.
machineRegistered	x		The user interface updates its list of registered machines.
machineUpdated	x		The user interface retrieves and displays updated details of a machine.
machineStateUpdated	x	x	The machine interface is notified of the changed state and acts in the order processing accordingly. The user interface retrieves and displays the updated state of a machine.

## Data Availability

Not applicable.
